# Flavin Catalysts in Organic Synthesis

**DOI:** 10.1002/anie.6683110

**Published:** 2026-07-17

**Authors:** Jan Freudenberg, Golo Storch

**Affiliations:** ^1^ Department of Chemistry Johannes Gutenberg University Mainz Mainz Germany

**Keywords:** biomimetic chemistry, flavin, homogeneous catalysis, isoalloxazine, photochemistry

## Abstract

Flavin cofactors are nature's all‐rounders with several essential roles in the active site of flavin‐dependent enzymes. These flavoenzymes display an astonishingly broad activity spectrum, which also translates to the organic laboratory. In synthetic chemistry, flavin catalysis is now an integral part of several organocatalytic and photochemical methods with applications ranging from total synthesis to biocompatible photoproximity labeling. Flavins take a unique position in the field: they are easily accessible through synthesis or biochemical methods, while additionally exhibiting orthogonal reactivity compared to other (organo‐)catalysts. Several flavin‐mediated organic reactions were developed in a biomimetic fashion guided by extensive literature on enzymatic mechanisms. Additionally, flavin modification to tailored catalysts enabled desired non‐natural transformations. This minireview gives an overview of and highlights the different key chemical transformations achieved with flavin catalysis in synthetic organic chemistry with a focus on photochemical oxidation and reduction, sensitization, oxygenation, and the activation of substrates by reversible covalent bond formation. The individual requirements for achieving specific flavin reactivity are illustrated based on example reactions. Finally, remaining challenges in the field of flavin catalysis are identified.

## Graphical abstract

Flavins and their congeners are potent catalysts for organic synthesis inspired by enzymatic transformations. In addition to catalysis in the flavin ground state via covalent intermediates, the excited singlet and triplet states unlock applications including photooxidation, photoreduction, and energy transfer via sensitization. This minireview focuses on organic transformations catalyzed by flavins developed within the last decade.

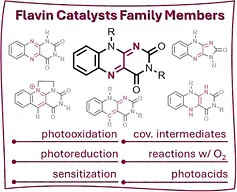

## 1 Introduction

Flavin cofactors are an integral part of active sites in several enzyme classes mediating a plethora of different chemical transformations (e.g., oxidation, dehydrogenation, monooxygenation, and reduction reactions) [1]. The unparalleled diversity of these reactions is part of the ongoing fascination for the small molecular organic cofactor. If achieved under controlled conditions in the organic laboratory, resource‐efficient and metal‐free methods result, rendering the development of flavin catalysis a venerable goal in modern organic synthesis.

The organic chemistry behind flavoenzyme‐catalyzed reactions has intrigued chemists for decades [2, 3]. In the early days, model systems mimicking the observed reactivity patterns were studied. This resulted in numerous publications regarding the synthesis of isoalloxazines and their derivatives. Most prominently, the redox properties of synthetic flavins have been systematically studied, and a substantial dataset is available today [4]. Ongoing trends involve late‐stage transformations or the design of new flavin derivatives [5]. Inter alia, site‐selective modification of flavins was explored as an alternative to synthesis from already functionalized small building blocks by Desage‐El Murr [6]. To tailor flavin absorptions and redox properties, the Baek laboratory designed pentacyclic flavin analogs, shifting their emission towards the deep‐red and near‐infrared [7].

The focus of this minireview is on catalytic applications of (tailored) flavins in organic transformations, particularly of new developments within the last decade. For a comprehensive overview of flavin catalysis, we refer to the textbook by Cibulka and Fraaije [8]. While recent review articles focus on specific areas of flavin catalysis, such as photochemistry [9, 10, 11] and biomimetic oxygenation [12, 13, 14], we aim for giving a broad overview of the synthetic applications. We have recently composed a systematic review on how modifications of flavin catalysts alter their catalytic properties [15]. In contrast, this minireview is structured according to individual transformations, which shifts the focus to applications in the organic laboratory: photochemical oxidation/reduction, sensitization, oxygenation, and the activation of substrates by reversible covalent bond formation as well as Brønsted acid photocatalysis are covered in that order. The (photo‐)catalysis with flavin‐dependent enzymes is not included as progress in this area has been reviewed elsewhere [16, 17, 18].

Throughout this minireview, the term “flavin” will be used for isoalloxazine heterocycles with general substitution patterns (Figure 1). Therefore, in line with the common notion in the field, the depicted catalysts are not always derivatives of the naturally occurring cofactor (–)‐riboflavin (vitamin B_2_) and, inter alia, do not necessarily share the N10‐ribityl substitution or the two C7‐ and C8‐methyl groups. We have also included the related quinoxaline‐based alloxazine compounds, which differ by the position of one substituent (*R*′′′). Since coenzyme F_420_ is also a naturally occurring cofactor [19], which is derived from the flavin family only by substitution of the N5‐nitrogen atom with a carbon atom, we included the deazaflavins in our discussion. Several modern photocatalysts, including pyrimidopteridines, share structural similarities with flavins but are not covered in this review [20].

**FIGURE 1:**
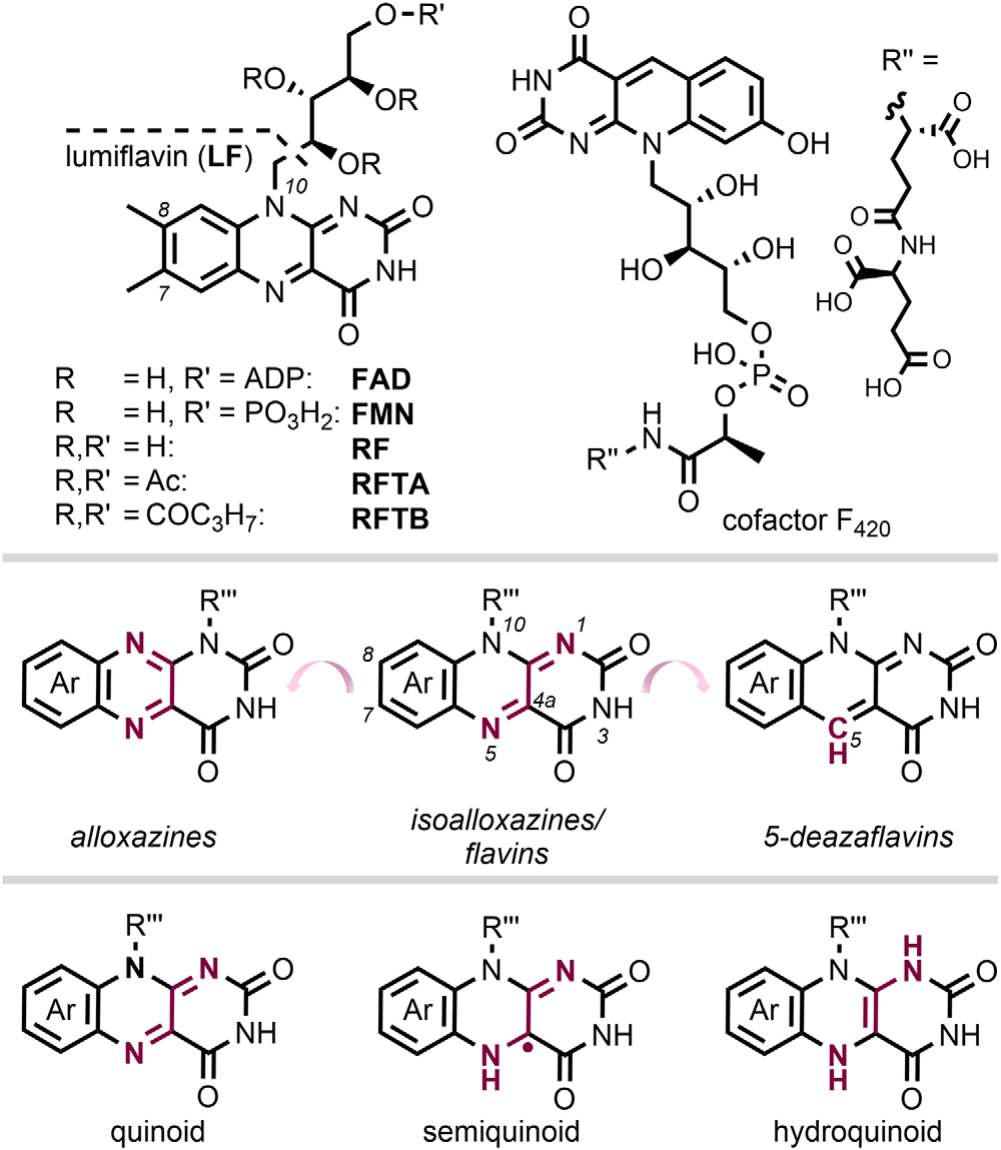
Core structure of substituted flavins and related compounds (**FAD** = flavin adenine dinucleotide, **FMN** = flavin mononucleotide, **RF** = (−)‐riboflavin, **RFTA** = (−)‐riboflavin tetraacetate, **RFTB** = (−)‐riboflavin tetrabutyrate).

The vast majority of catalytic cycles discussed in this minireview rely on the differing reactivity of catalyst oxidation states as illustrated for flavins in Figure 1 (bottom). Electron uptake of the oxidized, quinoid form, employed for photooxidations and sensitizations, furnishes the mono‐reduced, semiquinoid, and structurally elusive radical or the fully reduced hydroquinoid form, both main actors of photoreductions prepared mostly in situ. Additionally, N1‐alkylation of quinoid flavins increases their electrophilicity, improving covalent adduct formation in the ground state.

## 2 Photochemical Oxidation via Radical Intermediates

Among the various applications of flavin catalysts in photochemistry, oxidation with their excited quinoid form is by far the most prominent mode of action. The underlying reactivity comprises the formation of carbon‐centered radicals: The latter are either generated directly at the benzylic/allylic/α‐ether positions or are formed after initial heteroatom (e.g., N or S) or aromatic oxidation, respectively.

The oxidation of anisyl alcohol (**3**) to 4‐anisaldehyde (**5**) serves as a particularly well‐studied system (Figure 2). It proceeds via generation of carbon‐centered, benzylic radical **4** with photochemically excited flavin **1** [21, 22]. The latter is ultimately converted into the hydroquinoid form, which is re‐oxidized by O_2_ to close the catalytic cycle. Detailed photochemically induced dynamic nuclear polarization (photo‐CIDNP) studies by Gschwind revealed the dependence of the reaction mechanism on the solvent mixture—it controls whether two quinoid flavins are involved as electron acceptors generating substantial amounts of the flavin semiquinone, or whether one catalyst molecule accepts both electrons [23]. A recent addition to the toolbox of oxidative functionalization of benzylic positions stems from Cibulka. Deazaflavinium salt **2** facilitates the oxidation of benzylic alcohols in the absence of molecular oxygen [24]. The photocatalyst is re‐oxidized by reduction of the solvent acetonitrile, which in turn forms imine **8** and hydrolyzes to acetaldehyde (**9**). The combination with electrochemistry allows conducting the reaction in a chemoselective fashion, where thioethers are fully tolerated [25]. König and others disclosed that direct benzylic oxidation of the corresponding electron‐rich 4‐methylanisole (**10**) also leads to 4‐anisaldehyde (**5**) [26, 27], a reaction that can be expanded to electron‐deficient substrates using flavinium salt catalysts [28, 29, 30, 31, 32]. This reaction proceeds via benzylic radical **11**, generated by single‐electron transfer and deprotonation. Oxygen is required as a flavin oxidant and as an oxygen atom source. Details of the reaction mechanism were elucidated by Cibulka and Roithová based on electrospray ionization mass spectrometry [33]. Flavin‐mediated oxidation of benzylic positions was used as a strategy for photochemical polystyrene degradation [34].

**FIGURE 2:**
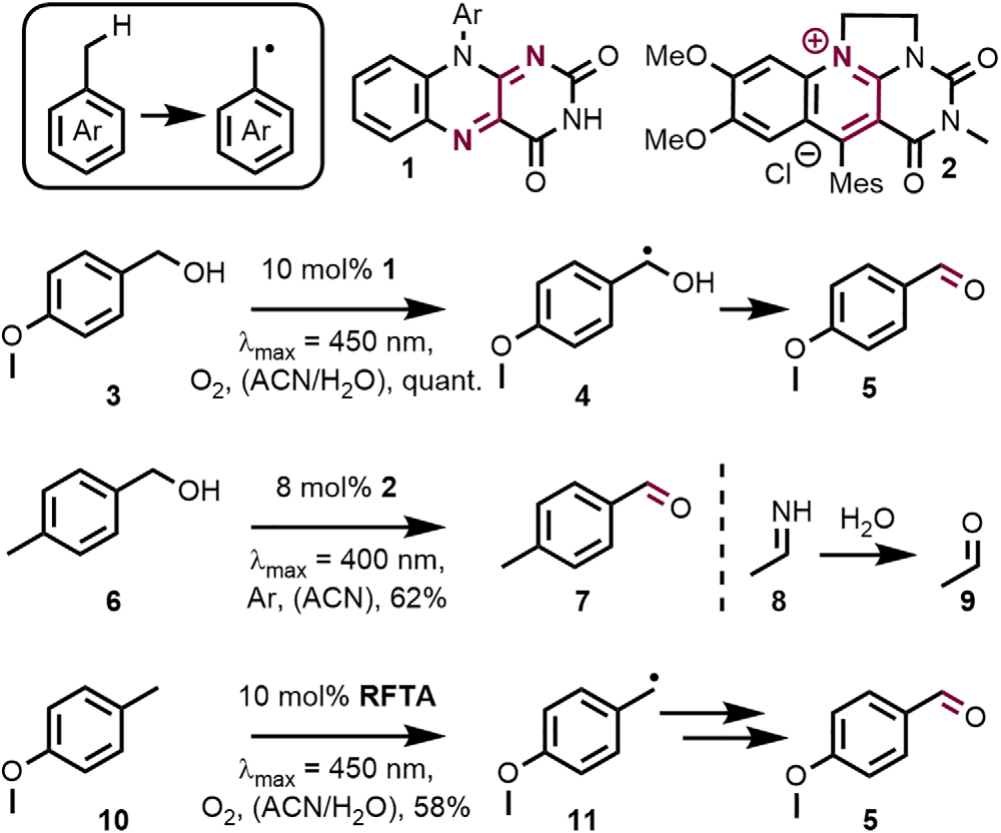
Benzylic oxidations catalyzed by flavins and dezaflavinium salts.

The generation of carbon‐centered radicals is not limited to benzylic positions. We recently disclosed silyl enol ethers **12** as equally competent substrates and reported their oxidation to the corresponding allylic radical species (Figure 3) [35]. In the presence of K_2_S_2_O_8_ as a sacrificial oxidant, the resulting products are unsaturated ketones **13** – a photochemical Saegusa‐Ito reaction occurs. Mechanistically, the reaction likely proceeds via a similar sequence of oxidation and deprotonation. This allows intercepting the carbon‐centered radical with suitable reaction partners: Addition of electron‐deficient olefins such as benzylidenemalonodinitrile (**16**) leads to ketone **18** after initial C─C bond formation with silyl enol ether **15**. The remarkable stability of the flavin catalysts also enables using the same catalyst in a sequential one‐pot synthesis. Following this strategy, the addition of CF_3_ radicals generated by oxidation of Langlois's reagent led to γ‐trifluoroketone **19**. This reactivity is not limited to silyl enol ethers: β‐ketoacid **20** is also converted to the α,β‐unsaturated ketone **21** by oxidation and decarboxylation, either stoichiometrically or catalytically with K_2_S_2_O_8_ [36]. Mechanistically, the reaction may either proceed via an oxidation of the enol tautomer or via oxidation of the carboxylic acid (vide infra). The reactivity resembles the activity of the EpxF enzyme, which is responsible for installing the α,β‐epoxyketone moiety in the proteasome inhibitor epoxomicin [37, 38].

**FIGURE 3:**
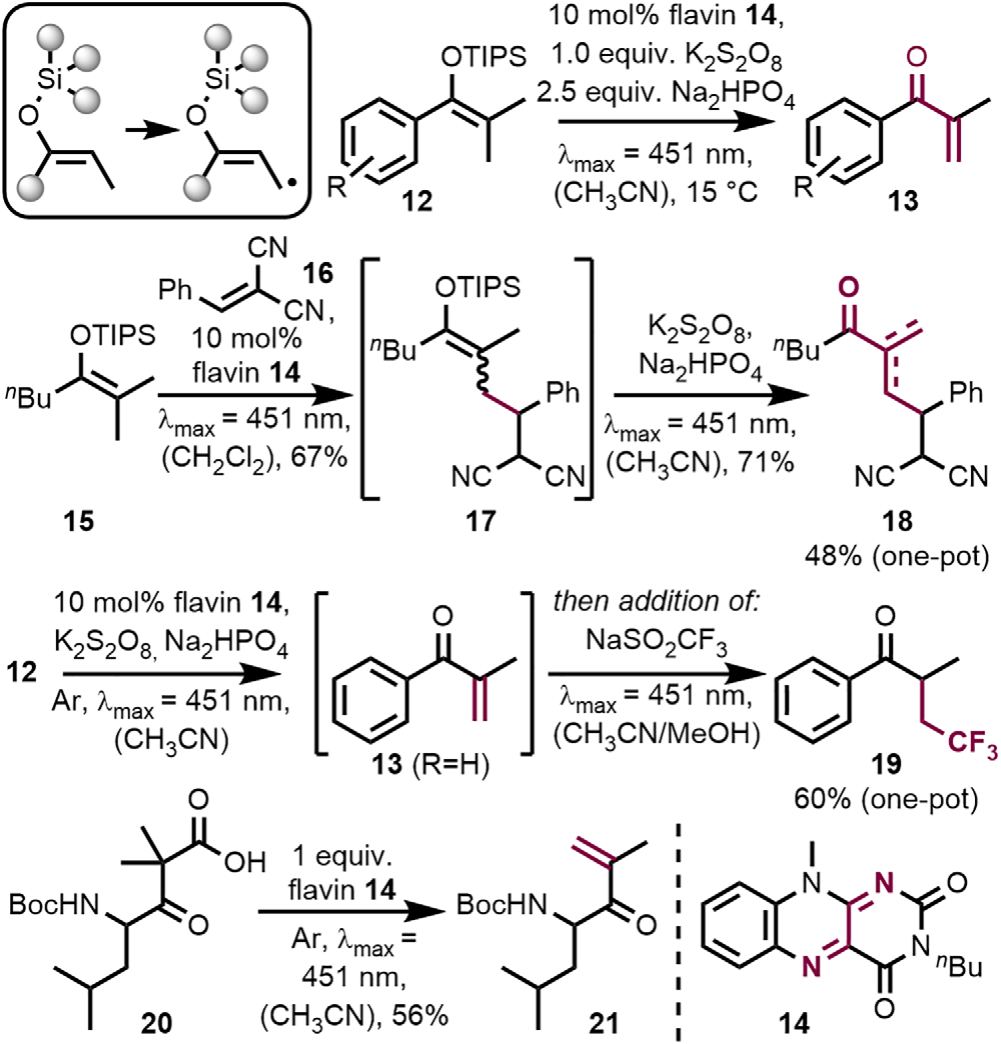
Oxidation of silyl enol ethers.

Carbon‐centered radicals are also accessible from dienes such as cycloheptadiene (**23**) and dehydroamino acids (e.g., **26**), either directly or after initial oxidation of the nitrogen position (to radical **28**). In both cases, the resultant radicals form covalent adducts, such as **24** with flavin catalyst **22**, which are reversibly cleaved (Figure 4). The intermediacy of radicals was demonstrated by their reaction with the persistent radical TEMPO at the carbon center, furnishing alkoxamine products (**25** or **27**). With dehydroamino acids, this procedure leads to unusual acylimines such as **29a** and **29b**. The latter are competent electrophiles for a variety of subsequent reactions and allow diversification towards unnatural amino acid side chains. It is reasonable to assume that the formation of covalent adducts such as **24** is not exclusive to the radical reactions described in Figure 4, but rather they are general (fleeting) intermediates, if steric demand permits their formation.

**FIGURE 4:**
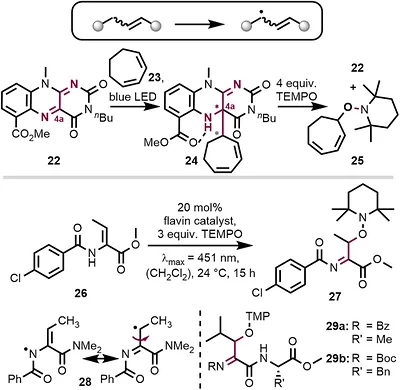
Oxidation of dienes and dehydroamino acids (TMP = 2,2,6,6‐tetramethylpiperidin‐1‐yl) via intermediately formed covalent adducts.

Photochemically excited flavins are also competent in oxidizing amines. Typically, the immediate products of these reactions are well‐studied nitrogen‐centered radical cations [39, 40]. Deprotonation then leads to the neutral carbon‐centered radicals, which engage in subsequent reactions. Among the established transformations are the oxidation of benzylamines to imines, utilized in the synthesis of benzimidazoles [41]. With tertiary amines such as **30**, the oxidation and deprotonation sequence leads to iminium cations **32** via neutral carbon‐centered radicals **31**, which readily deprotonate to enamines **33**. This strategy has been applied by Wasa for the covalent tagging of biologically active natural products containing a piperidinyl substituent (Figure 5) [42]. When oxidized to the corresponding enamine, this position allows a clean reaction with tetrazines, leading to adducts **36**—a consequence of Diels‐Alder addition, N_2_‐, and β‐elimination. The authors noted significantly increased product yields when using C6‐Br flavin **35**, attributed to its higher excited state oxidation strength compared to the parent C7,C8‐dimethyl congener. The desaturation with flavin catalysts was also used by Wennemers, who disclosed conditions for converting carbamates **37** under two different reaction conditions [43]. With cesium carbonate as a base, the pH of the reaction mixture remains at 7 or higher, leading to hydration and the formation of the hemiaminal **38**. In contrast, with lutidine, the β‐position is deprotonated, and enamine **39** forms. The added potassium peroxodisulfate regenerates the flavin catalyst by oxidation.

**FIGURE 5:**
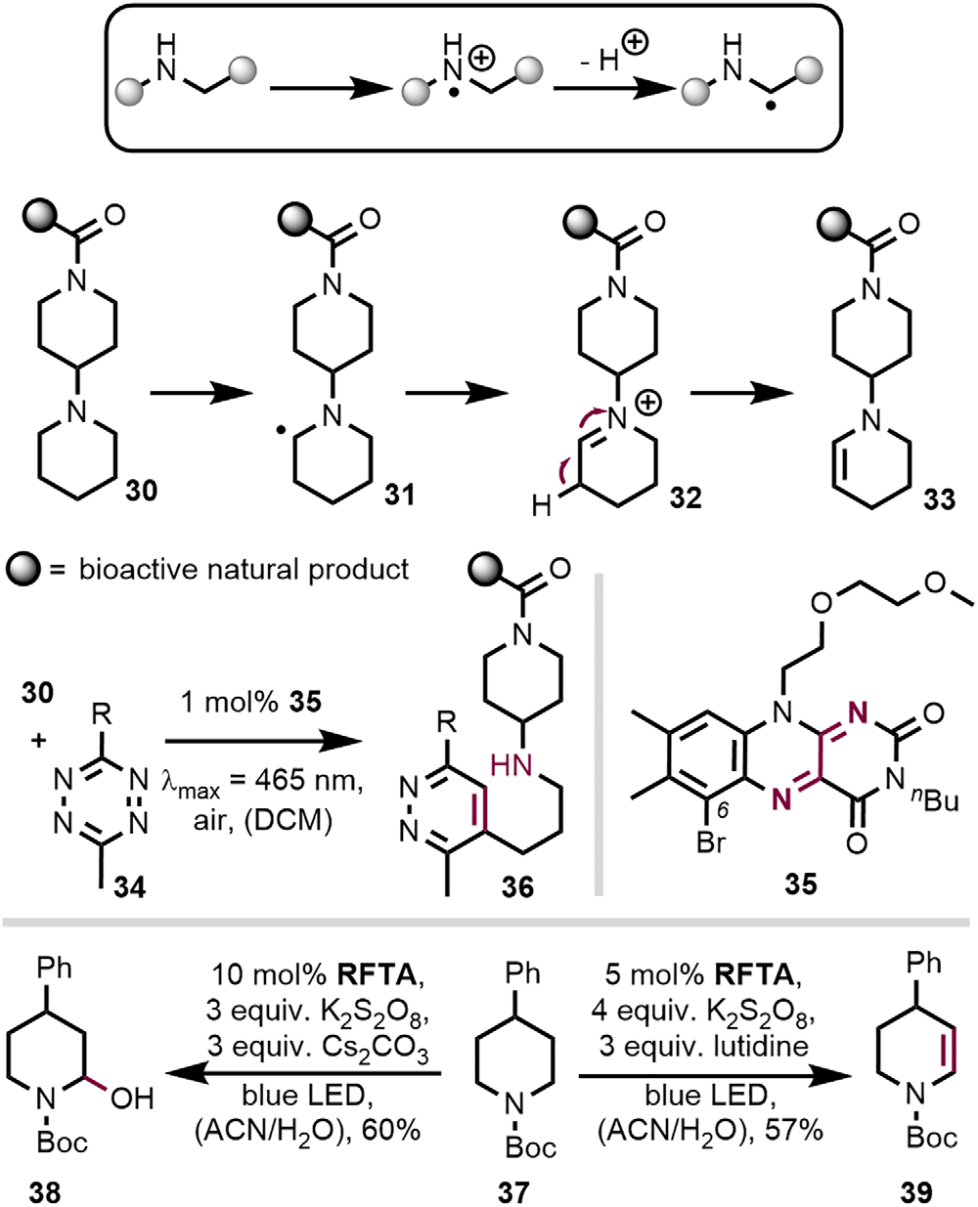
Flavin‐catalyzed amine oxidations.

The initial formation of N‐centered radicals is also achieved with enamines that are generated in situ from aldehydes in the presence of secondary amines. Arakawa and Imada demonstrated that enamines are oxidized by excited flavin catalysts. The resulting radical species are trapped by TEMPO, leading to a net α‐oxidation [44]. In another study by Cibulka, the initial hemiaminal is oxidized, which leads to the conversion of aldehyde substrates into amide products [45].

Indoles (e.g., **40**) and azaindoles are oxidized to their spin‐delocalized cations [46] after single‐electron transfer using lumiflavin photocatalysis. Bloom and co‐workers achieved the subsequent selective C3‐functionalization through the introduction of water as a transient protection group at the C2‐position—the hydroxylated radical intermediates then react at the C3‐atom. Trapping with an appropriate radical acceptor such as dehydroalanine **41** furnishes unnatural amino acid residues (e.g., tryptophan derivative **42**) after elimination of water (Figure 6) [47].

**FIGURE 6:**
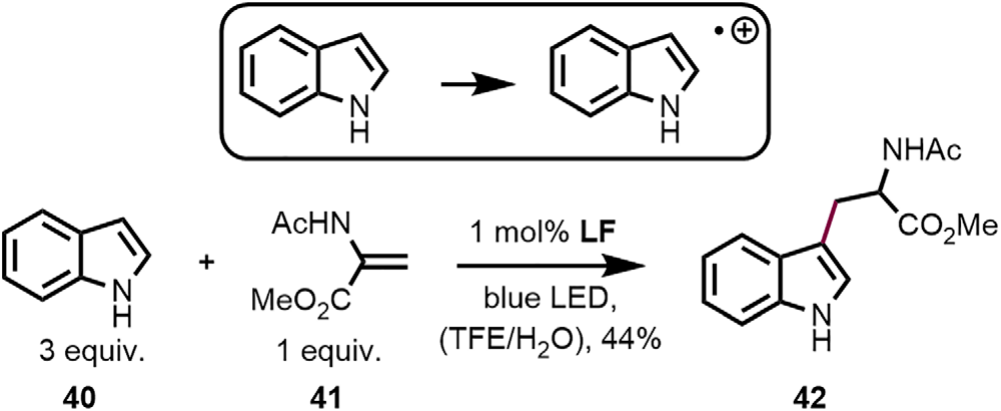
Indoles as sources of radicals and their reactions at the C3 position.

Carbon‐centered radicals adjacent to sulfur atoms are generated by flavin‐mediated oxidation of thioethers (Figure 7, left). Both MacMillan and Procter successfully applied this strategy to activate a variety of cyclic and acyclic thioethers, including dimethyl sulfide, using (−)‐riboflavin [48, 49]. In combination with dehydroamino acid substrates such as **43**, this method accesses non‐proteinogenic amino acids and methionine derivatives (e.g., **44**). Mechanistically, the authors provide evidence for an initial single electron oxidation to a sulfur‐centered radical cation followed by the deprotonation to the neutral carbon‐centered radical. The latter can also be trapped with alkenes, alkynes, or nitriles, exemplified by the reaction of 1,2‐bis(phenylsulfonyl)ethylene **45** to olefin **46** [50].

**FIGURE 7:**
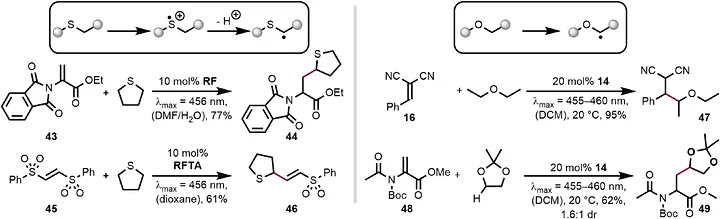
Alkyl/cycloalkyl thioether versus ether/acetal oxidation for the generation of carbon‐centered radicals.

Excited quinoid flavin catalysts also enable the oxidation of thiourea at the sulfur position, which leads to thiyl radicals after deprotonation. These species are competent in abstracting hydrogen atoms adjacent to substrate hydroxy groups. The resulting C‐centered radicals reduce the flavin catalyst to the hydroquinone. This strategy was used by Lin for the conversion of aliphatic alcohols into the corresponding carbonyls—the authors applied electrochemistry for the re‐oxidation of the reduced catalyst [51].

Sulfur heteroatom oxidation is also at work in the reaction of thiols with amines to sulfin‐ and sulfonamides using excited **RFTA**. In the proposed catalytic cycle, a photooxidized disulfide intermediate is attacked by an amine nucleophile. A thioamine radical cation results, which is further oxidized by molecular oxygen concomitant to regeneration of the flavin catalyst [52].

Carbon‐centered radicals adjacent to oxygen atoms offer conceptually similar opportunities for follow‐up chemistry. Since ethers are significantly more difficult to oxidize, the mechanism most likely involves a hydrogen atom abstraction. Our group has explored this avenue by activating a series of cyclic and acyclic ethers, acetals, and ketals as substrates, which were efficiently trapped by benzylidenemalonodinitrile (**16**) to α‐substituted ethers **47** (Figure 7, right) [53]. Addition of the carbon‐centered radical of 2,2‐dimethyldioxolane allows functionalization of dehydroamino acids such as **48** to protected non‐natural analogs (e.g., **49**). Mild acidic deprotection leads to the non‐proteinogenic amino acid 4,5‐dihydroxynorvaline (Dnv).

The oxidation of phenols [54] and naphthols to their spin‐delocalized aryloxy radicals was successfully explored by the groups of König, Pathak, and Sarkar (Figure 8) [55, 56, 57]. While the intermediate formation of open‐shell species such as **52** from naphthol derivative **50** is confirmed by TEMPO‐trapping experiments, two mechanistic alternatives were explored for the subsequent reactivity: aryloxy radicals either directly engage in bond‐forming steps or are oxidized again to form a carbocation. The latter serves as an electrophile for intramolecular attack (as in the case of naphtho[2,1‐*b*]furan derivative **51**). The generation of aryloxy radicals was also used by Oslund, Fadeyi, and Frenette for the proximity labeling of tyrosine residues—radical‐radical recombination furnishes the adducts **55** after final oxidation [58]. MacMillan and coworkers succeeded in coupling these radicals to phenoxazines [59].

**FIGURE 8:**
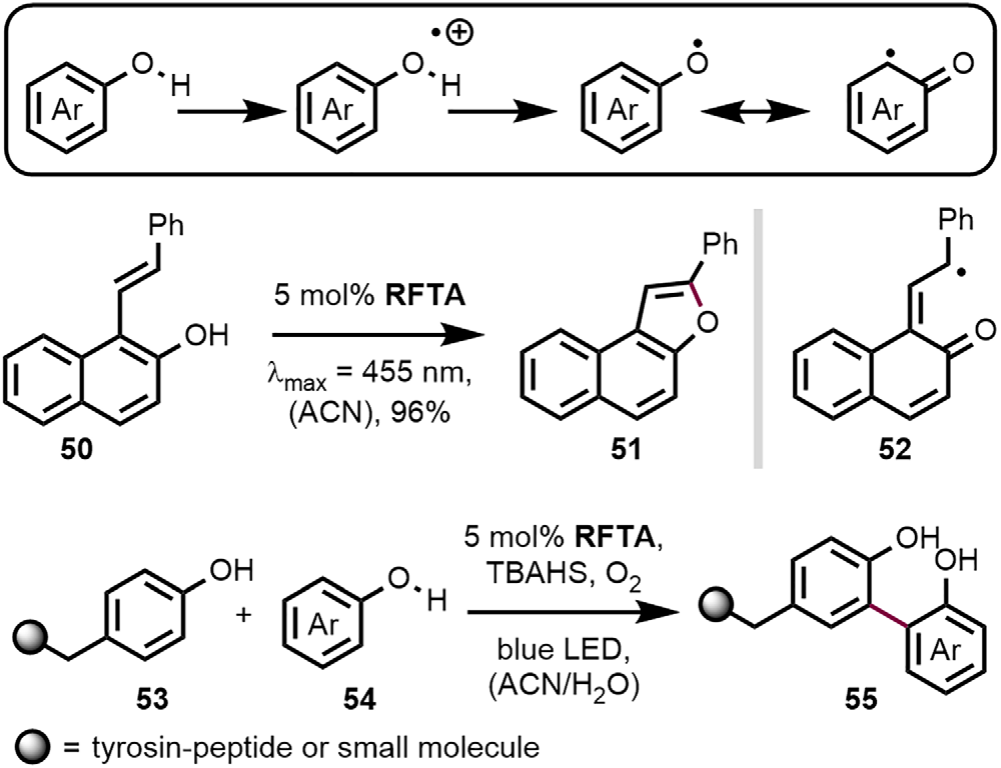
Inducing carbon‐centered radical reactivity in phenol derivatives (TBAHS = tetrabutylammonium hydrogen sulfate).

Another oxidative strategy for obtaining C‐centered radicals with flavin catalysts starts from carboxylic acids. Similar to the flavoenzyme fatty acid photodecarboxylase [60, 61], excited molecular flavins generate the corresponding radical species upon release of CO_2_ (Figure 9). The reactive intermediates can be trapped by diethyl 2‐ethylidenemalonate (**57**). The MacMillan group developed this strategy and succeeded in selectively modifying peptides exclusively at the C‐terminus due to the differences in oxidation potentials of terminal versus internal carboxylates. The method was successful for several peptides with C‐terminal alanine residues **56** [62]. The high level of specificity for C‐terminal carboxyl groups in the presence of glutamic acid and aspartic acid allowed obtaining modified analogs **58** and application of the decarboxylation strategy in proteomics [63]. The Wang group observed a special case with Fmoc‐protected amino acids (e.g., **56**'): **FMN**‐mediated generation of the C‐centered radicals led to their subsequent quenching with oxygen [64]. This sequence allowed for isolation of the corresponding hemiaminal **59**. Aliphatic carboxylic acids were employed as alkyl radical sources in decarboxylative allylation reactions catalyzed by excited **RFTA**. Additions to allyl/benzothiazol sulfones such as **60** and subsequent radical fragmentation furnish alkenes or benzothiazoles (e.g., **62**), respectively [65]. In contrast, the carboxyl radical generated from biphenyl carboxylic acid **63** undergoes intramolecular C─O bond formation to coumarin **64** faster than decarboxylation [66]. In combination with Selectfluor, flavin catalysis enabled the decarboxylative fluorination of several (polymeric) carboxylic acids [67, 68, 69]. The cyanation of carboxylic acids was accomplished by Gonzalez‐Gomez with TsCN [70].

**FIGURE 9:**
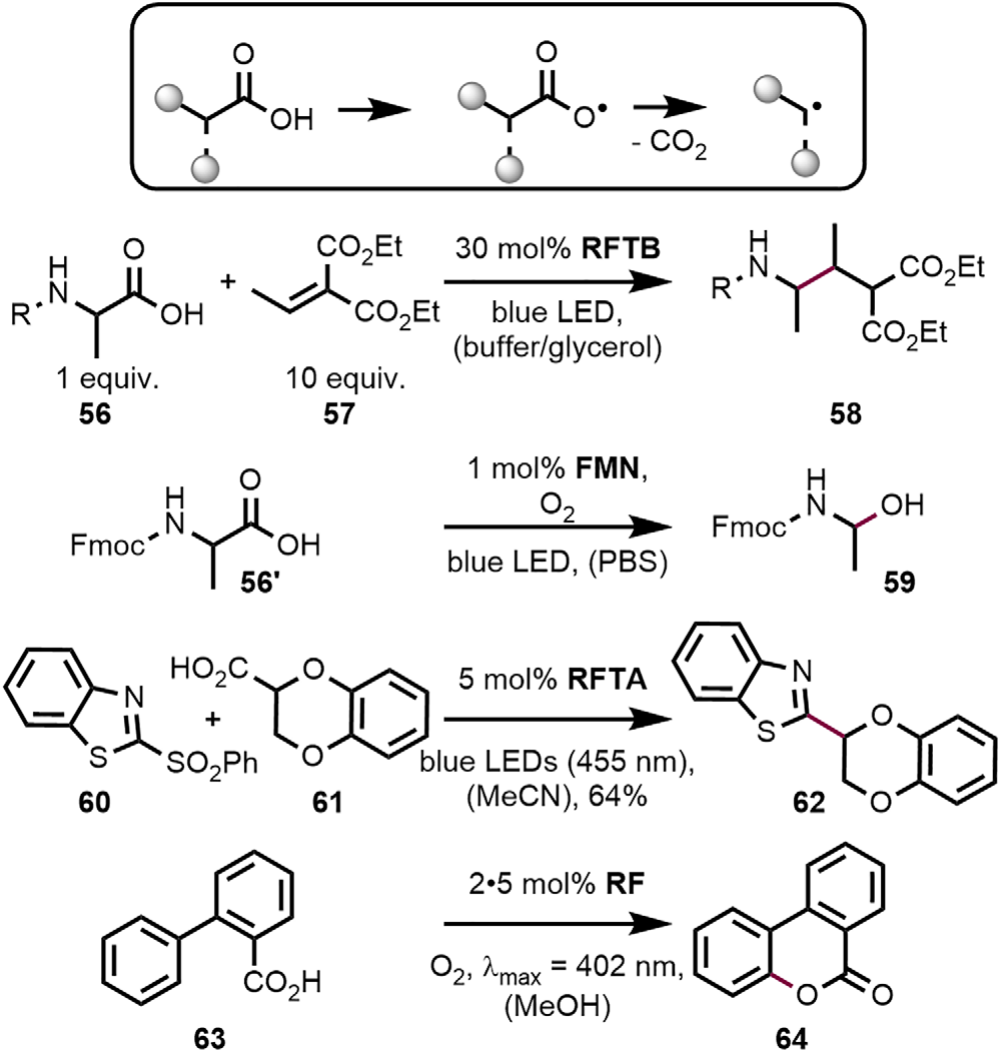
Carboxylic acids as sources for C‐centered radicals.

The Bloom laboratory accessed C‐centered radicals from boronic acids (e.g., **65**, Figure 10) via oxidation with lumiflavin catalysts and realized C─C bond formation with electron‐deficient olefin **57** to malonate **66** [71]. These reactions are compatible with a large variety of aromatic boronic acid substrates. They are conducted in an aqueous buffer, where the boronic acids are activated as ate complexes prior to oxidation. With dehydroamino acids as reaction partners for the C‐centered radicals, unnatural amino acid residues are incorporated into peptides **67** [72].

**FIGURE 10:**
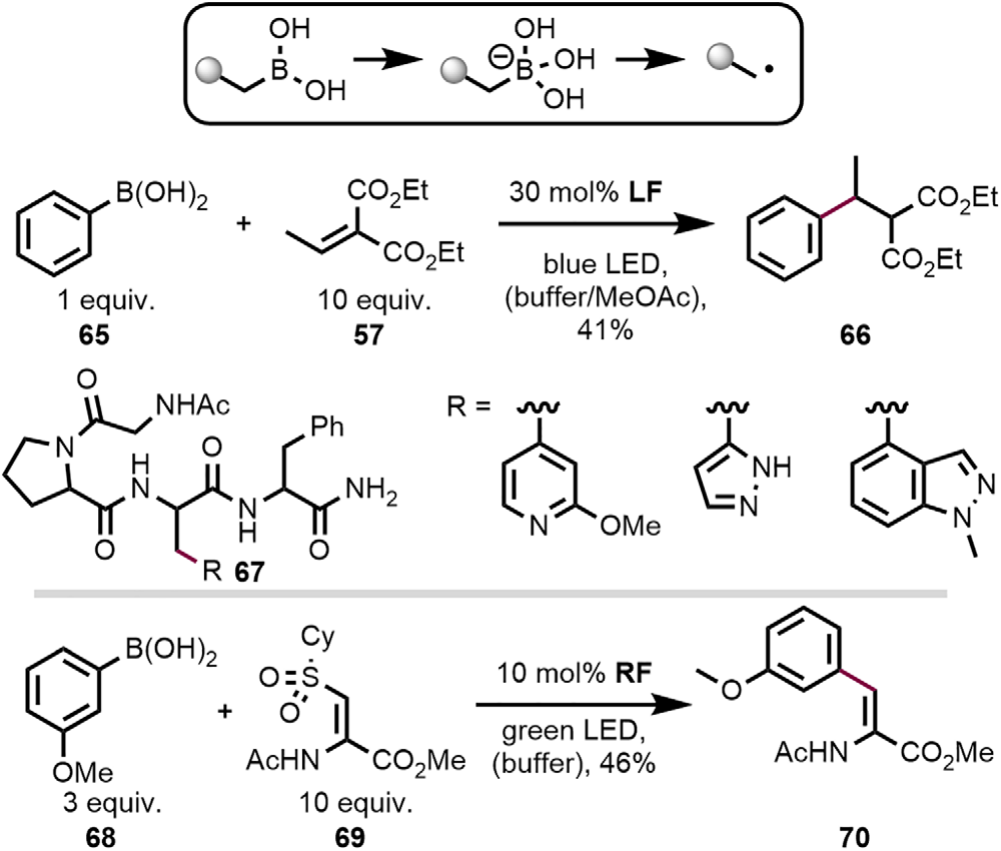
Boronic acids as sources for C‐centered radicals.

For aromatic boronic acids, the combination of flavin catalysis and palladium complexes is ideal: the latter significantly increases the efficiency of the addition step due to intermediate formation of palladium aryl species [73]. With β‐sulfonyldehydroamino acid **69**, the radical addition step is followed by sulfinate elimination. The modified dehydroamino acid **70** results when using boronic acid **68** [74].

## 3 Photochemical Reduction

Reductive flavin catalysis requires excitation of the hydroquinoid (e.g., **71**, Figure 11) or semiquinoid catalyst state. The natural pendant and inspiration to many laboratory reactions stem from the DNA photolyase. This enzyme utilizes the excited reduced flavin cofactor as a single‐electron reductant to induce cycloreversion of the thymine dimer, thereby repairing photodamaged DNA [75, 76].

**FIGURE 11:**
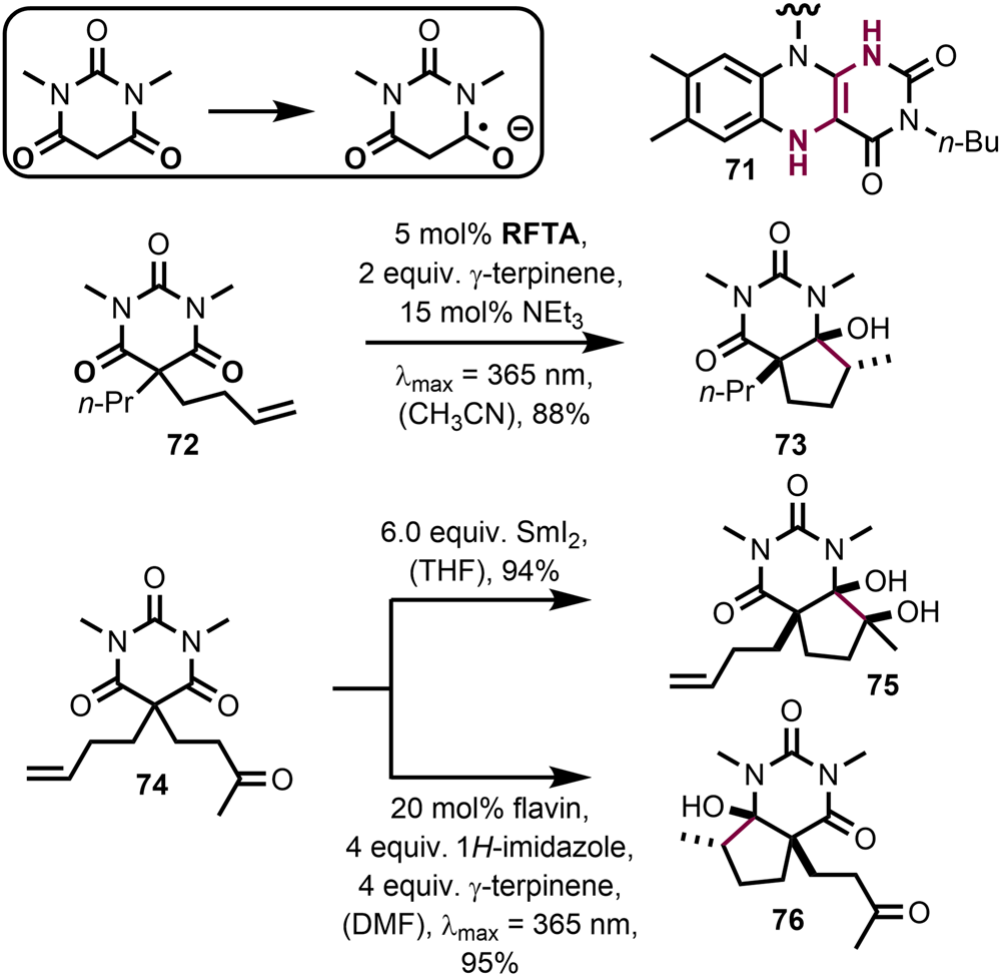
Reductive cyclization of barbiturates.

In the organic laboratory, our group achieved a similar reactivity using γ‐terpinene as a reductant for excited quinoid flavins, generating their hydroquinoid state. Upon catalyst excitation, barbiturates such as **72** are reduced to ketyl intermediates. A subsequent 5‐*exo*‐*trig* cyclization furnishes bicyclic hemiaminal **73** [77]. This reactivity complements reductive cyclizations with samarium(II) iodide [78, 79]: While pinacol coupling to diol **75** is induced by SmI_2_ for barbiturate **74** carrying alkyl ketone and ω‐alkenyl substituents, this hybrid substrate is chemoselectively cyclized to hemiaminal **76** utilizing flavins [80].

Photochemical reduction is also achieved with deazaflavin catalysts (Figure 12). Kutta, Cibulka, and König disclosed the formation of the semiquinone radical state by reduction of the excited catalyst **77** with a tertiary amine (here: DIPEA). Re‐excitation of the radical leads to a strongly reducing species that is capable of converting aryl halides (e.g., aryl bromide **78**) into the corresponding aryl radicals, followed by hydrogen atom abstraction to dehalogenated aromatic product **79** [81]. Similar tailored deazaflavin catalysts even dehalogenate aryl chlorides efficiently (**80**–**82**) [82]. The same reduction strategy is successful for deprotecting sulfonamides into amines [83]. Interestingly, Cibulka and Sikorski discovered that reductive catalysis is also possible with deprotonated alloxazine catalysts **83**. Similarly, the excited anion is a competent reductant for aryl halides, including 4‐bromobenzonitrile (**84**). The γ‐terpinene reagent serves as the terminal reductant, and benzonitrile (**85**) is obtained [84]. A combination of both concepts uses deprotonated deazaalloxazine catalysts with enhanced reductive strength [85].

**FIGURE 12:**
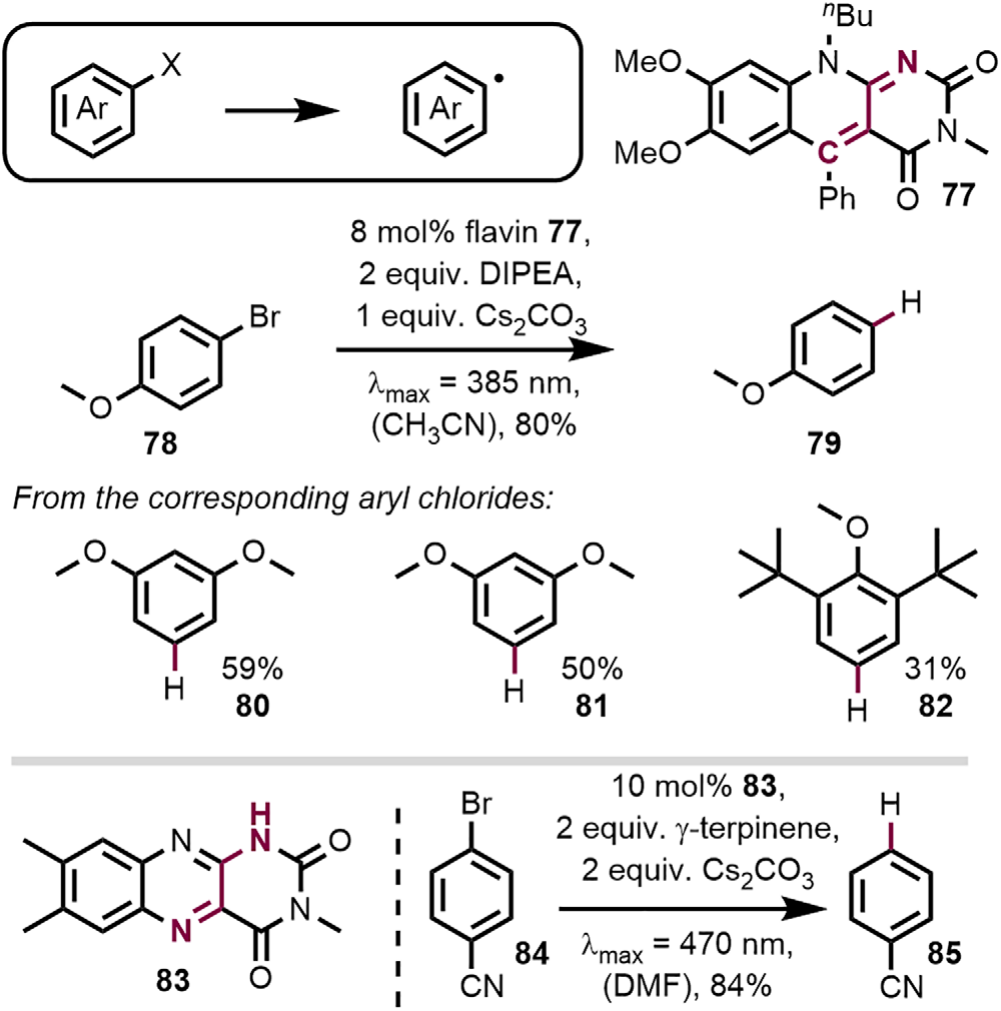
Reductive dehalogenation of aryl halides.

## 4 Triplet Energy Transfer

Excited flavins undergo intersystem crossing to their triplet state (triplet energy of riboflavin/lumiflavin: *E*_T_ = 209 kJ mol^−1^) [86]. The activity of flavins as triplet sensitizers was described by Radda as early as 1967, who reported that excitation of lumiflavin in the presence of retinol leads to its *cis/trans* isomerization [87]. The Gilmour group achieved efficient isomerization of unsaturated ester (*E*)‐**86** to (*Z*)‐**86** with a catalytic amount of (−)‐riboflavin by sensitization (Figure 13) [88]. The triplet energy of flavins is sufficient to sensitize the (*E*)‐isomer, but too low for sensitization of the accumulating (*Z*)‐isomer with reduced conjugation due to allylic strain. This robust method (see examples **87–89**) tolerates extension to immobilized versions of the catalyst [89, 90, 91]. The flavin‐mediated olefin isomerization was also successfully applied in total synthesis [92]. With α,β‐unsaturated carboxylic acid **90**, olefin isomerization is followed by radical cyclization to coumarin (**91**) through subsequent oxidation of the carboxylate to the carboxyl radical (cf. Figure 7) [93]. Here, oxygen serves as the terminal oxidant for the hydroquinoid flavin.

**FIGURE 13:**
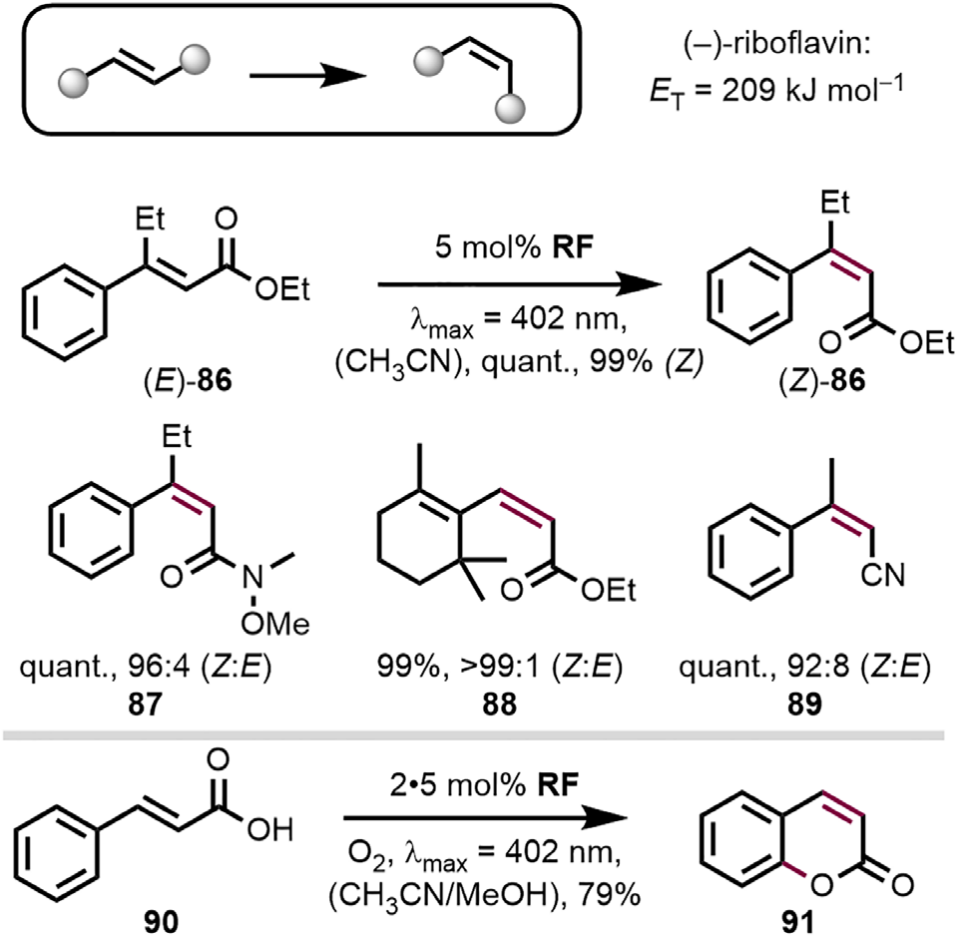
Olefin isomerization by triplet sensitization.

In many cases, including (conjugated) diene substrates, the triplet energy of naturally occurring flavins is too low to enable efficient energy transfer. However, this can be countered by tailoring the flavin catalyst, either by ring contraction to imidazolonequinoxalines, by switching to the alloxazine regioisomers, or by variation of the skeleton to deazaflavins. Flavin fluorination was also found to significantly affect the triplet quantum yield [94]. Our group disclosed flavin analog **92**, formed by a base‐induced ring‐contraction and subsequent release of CO_2_ (Figure 14). These catalysts are characterized by an increase in triplet energy of *E*_T_ = 244 kJ mol^−1^. As a consequence, sensitization reactions such as the *E/Z*‐isomerization of cycloheptadiene (*E*_T_ = 228 kJ mol^−1^, **23**) [95] followed by thermal cyclization [96] to bicycle **93** are facilitated [97]. Parent flavins do not yield the product. Ring‐contracted flavins are stable catalysts and were successfully applied in sequential reactions that required activity in both sensitization and hydrogen abstraction. Tropolone (**94**) is thus converted into bicyclo[3.2.0]hept‐3‐ene‐2,7‐dione (**95**), which is ring opened by methanolysis. Addition of the acyl radical generated by hydrogen atom abstraction from 4‐*tert*‐butylbenzaldehyde furnishes cyclopentanone products **96**.

**FIGURE 14:**
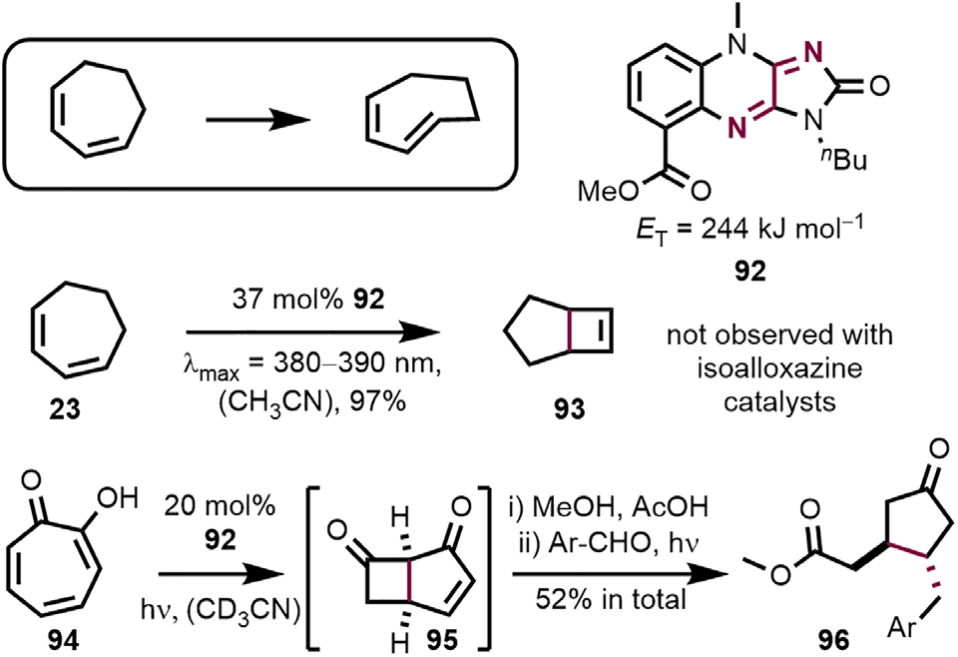
Ring‐contracted flavins as sensitizers with increased triplet energy.

Compared to flavins, alloxazines such as **97** exhibit triplet energies increased by approx. ∼40 kJ mol^−1^. Cibulka applied the latter as catalysts in [2 + 2]‐photocycloaddition reactions with tethered diene **98**, leading to bicycle **99** (Figure 15) [98].

**FIGURE 15:**
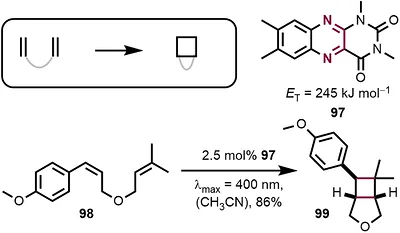
An alloxazine catalyst with increased triplet energy.

Deazaflavin **100** is also characterized by an increased triplet energy of *E*_T_ = 249 kJ mol^−1^. In model studies, the Hackenberger group showed that diazirine **101** (*E*_T_ ≥ 251 kJ mol^−1^) is successfully activated to the carbene intermediate (Figure 16), confirmed by the formation of secondary alcohol **102** [99]. The biocompatible deazaflavin catalyst allows photoproximity labeling of serum albumins with diaziridines such as biotin‐based derivative **103**.

**FIGURE 16:**
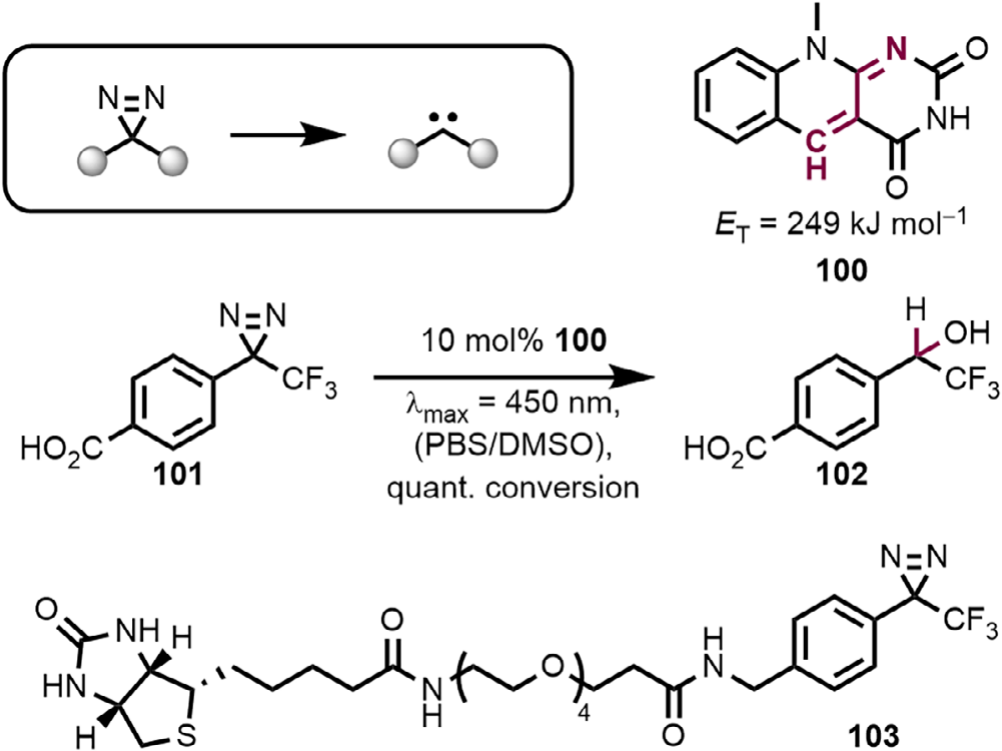
A deazaflavin as a biocompatible catalyst for photoproximity labeling (PBS = phosphate‐buffered saline).

## 5 Reactions with O_2_

Photosensitization of triplet oxygen for subsequent oxidation reactions is also feasible with flavin catalysts. The lowest‐lying singlet state of O_2_ is 94 kJ mol^−1^ above the triplet ground state [100] and is well accessible with flavin catalysts. As a consequence, reactive singlet oxygen can be generated. The groups of König and Sarkar used this property for activation of the benzylic position in phenol substrate **104** (Figure 17) [101]. Singlet oxygen initially abstracts the highlighted hydrogen atom and subsequently forms *para*‐quinone methide **105**, concomitant to the release of hydrogen peroxide. If methanol is present as a nucleophile, adduct **106** is obtained in good yields. A series of other nucleophiles such as aliphatic/aromatic thiols or N‐heterocycles is also compatible with the method. In cases of sensitive substrates, singlet oxygen generation is hampered by concurrent substrate oxidation. Our group developed modified flavin catalysts for these cases, which are less oxidizing in the photochemically excited state but are still capable of sensitization. With such an aminoflavin catalyst **108**, we achieved the Schenck ene reaction of silyl enol ether **12** to the corresponding hydroperoxide **107**, which is otherwise inefficient with unmodified isoalloxazines [35]. The converse effect—namely, avoiding the sensitization of oxygen—was reported as a result of aryl substitution at the flavin core [102].

**FIGURE 17:**
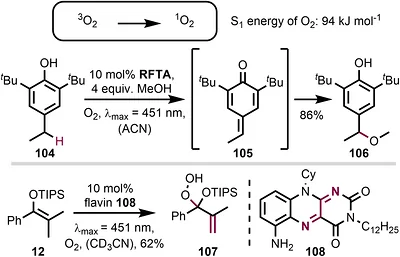
Singlet oxygen generation with flavins.

Many of the reactions discussed in Section 2 (*vide supra*) achieve reoxidation of the flavin catalyst by conversion of oxygen to hydrogen peroxide. This reactivity is well‐studied and proceeds via initial single‐electron reduction and formation of superoxide [103, 104]. In general, this catalyst property allows using O_2_ from air as a terminal oxidant also for other catalytically active species. A particularly interesting scenario is the combination of flavin and iodine catalysis: flavinium catalyst **109** regenerates the iodine catalyst by oxidation of iodide. The resulting hydroquinoid flavin **110** activates O_2_ and forms hydroperoxide **111**, which also oxidizes iodide to iodine. The Iida group studied this catalytic combination extensively, exemplified by the oxidative formation of 1,2,3‐thiadiazoles **114** from hydrazones **112** (Figure 18) via olefin **113** [105]. The group has applied this strategy to a variety of other iodine‐catalyzed reactions [106, 107, 108, 109, 110], including the sulfenylation of pyrazolone **115** to thioether **116** [111] and the selective C─N bond formation of N‐methylindole (**118**) with pyrazole (**119**) to its substituted analogue **120** [112]. In a related fashion, a dihydroalloxazine catalyst reduces oxygen, and the resulting alloxazine hydroperoxide is capable of sulfoxidation. Water elimination precedes electrochemical regeneration of the alloxazine radical, followed by regeneration of the dihydroalloxazine catalyst through disproportionation [113]. A conceptually related approach relying on the oxidation of chloride to chlorine radicals was used to spawn hydroperoxyl radicals in the presence of molecular oxygen, which allows the conversion of internal alkynes into 1,2‐diketones [114].

**FIGURE 18:**
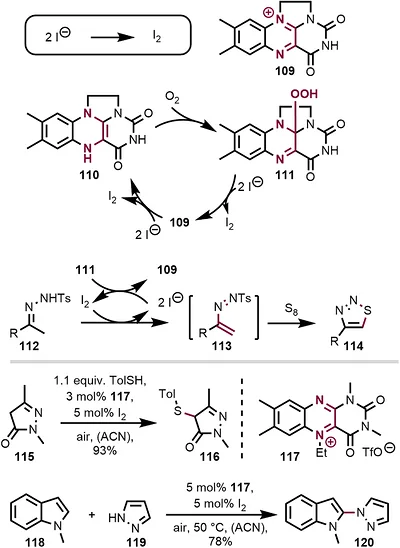
The combination of flavin and iodine catalysis.

In nature, flavin‐dependent halogenase enzymes also depend on hydroquinoid flavins [115]. Here, the reduced cofactor reductively activates O_2_ forming a flavin hydroperoxide intermediate. The latter converts a halide into the corresponding hypohalite, an electrophilic species capable of substrate halogenation [116, 117, 118]. The groups of Wolf and König developed a chlorination strategy based on this mechanism: hydrochloric acid acts as a source of chloride, which is turned into electrophilic Cl‐species (Figure 19) [119]. After photochemical excitation of the flavin catalyst, anisyl alcohol (**3**) converts the catalyst to the hydroquinoid state. A reaction with O_2_ follows, and the released hydrogen peroxide is trapped by acetic acid as its peroxy acid **123**. The latter generates hypochlorite from chloride. The electrophilic chlorination of substrates, including anisole (**121**) to halogenated analog **122**, follows. In a similar approach, our group reported the bromination of phenol substrates including tyrosine **124**, to the brominated amino acid **125** with a modified bisflavin catalyst **126** and lithium bromide [120]. Lutidinium oxalate (‘*LutOx*’) proves critical as a reductant for the photochemically excited flavin catalyst, since others lead to unproductive consumption without substrate conversion. Flavin catalysts are also compatible as co‐catalysts with vanadium‐dependent peroxidases to achieve electrophilic halogenation reactions [121].

**FIGURE 19:**
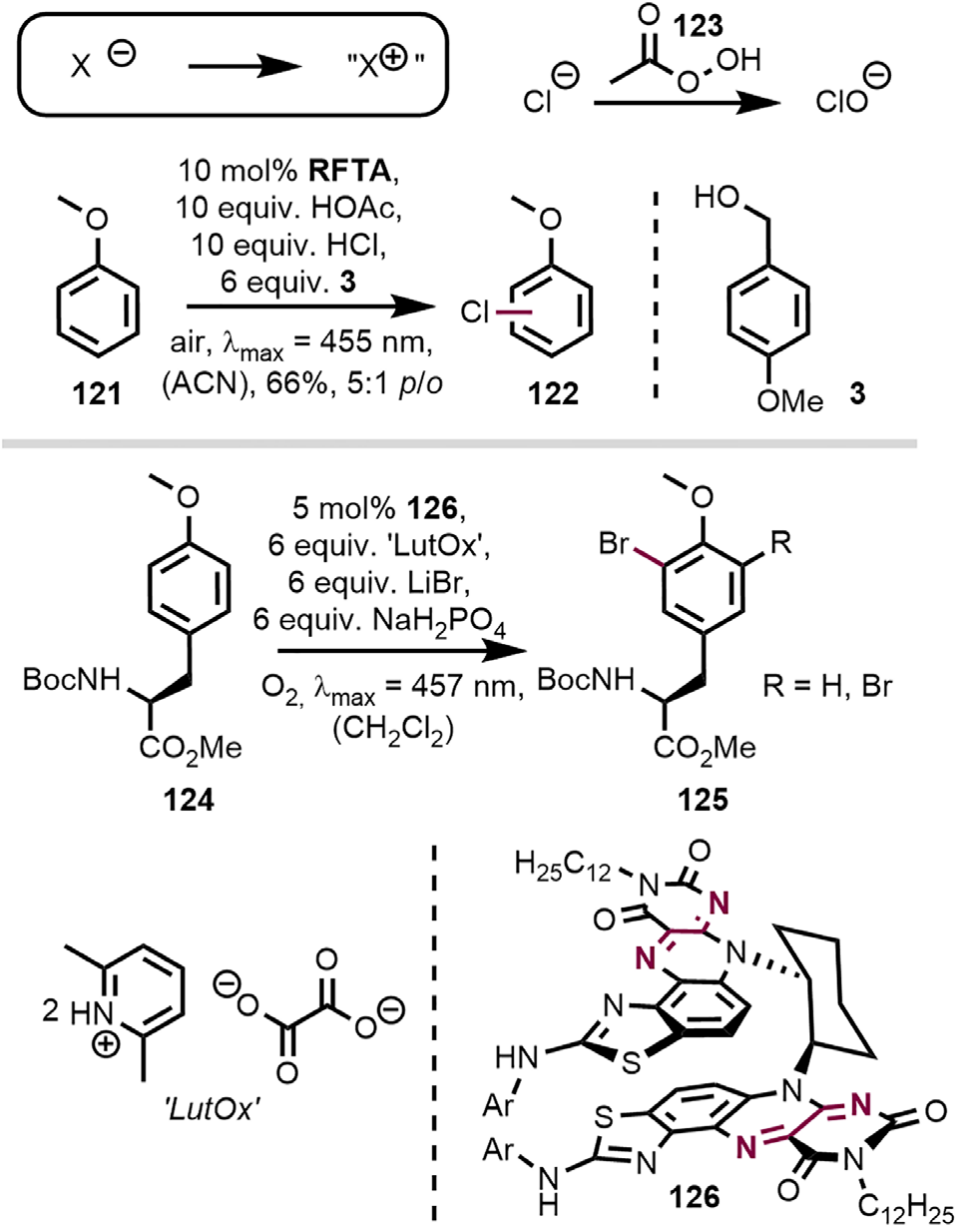
Halogenation using flavin catalysts.

## 6 Catalysis via Covalent Intermediates

Enzyme mechanisms with flavin cofactors often rely on the intermediate formation of covalent bonds between the cofactor and a substrate/reagent. Synthetically, several reductants for flavins add to the cofactor to form an intermediate adduct, which releases the hydroquinone and the oxidized reagent. In early studies by Naota and Imada, diimide was generated through this mechanism from hydrazine for the reduction of olefin substrates [122, 123, 124, 125]. The Cibulka group systematically investigated adduct formation with different nucleophiles (Figure 20) [126]. The authors developed a method for adding carbon nucleophiles to flavinium salt **127**, which enables the use of nitroalkanes such as nitroethane (**128**) for the C–C bond formation to secondary alcohol **129** by a Henry reaction [127]. The concept relies on an initial covalent adduct **130**, which releases nitrite to form the active electrophile **131**. With water as solvent and nucleophile, the aldehyde is released by a formal Nef reaction. Oxygen then reoxidizes the catalyst. The Nef reaction with flavinium salts and nitroalkanes was also used by Foss Jr. for the generation of nitrites and as an aerobic method for regenerating TEMPO in alcohol oxidation [128]. The addition of carbon nucleophiles to flavins is broadly applicable and includes ester **132** [129]. Here, the deprotonated substrate forms an adduct with flavin **134**. The carboxylate is released as a leaving group, which offers a deprotection strategy for esters, as demonstrated by obtaining carboxylic acid **133**.

**FIGURE 20:**
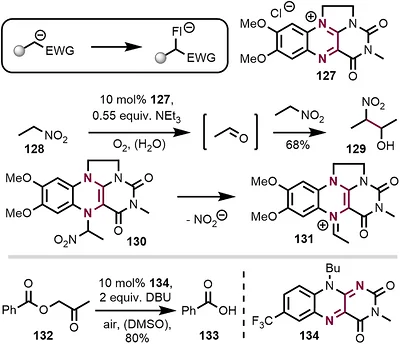
Flavin catalysis via covalent adducts with carbon nucleophiles unlocks applications in Henry reactions (top) and the deprotection of carboxylic acids (bottom).

Phosphorous nucleophiles also form reactive adducts with flavinium salts (Figure 21). Cibulka and coworkers observed that triphenylphosphine efficiently reacts with flavinium salt **135**. A catalytic Mitsunobu reaction can be realized: The flavinium catalyst substitutes the azodicarboxylate, exemplified by the ester formation (**137**) between benzoic acid (**133**) and benzyl alcohol (**136**) [130]. Mechanistically, the reaction of the latter with adduct **138** leads to hydroquinoid flavin **139**, which is regenerated by oxidation with air. Naturally occurring (−)‐riboflavin is also a competent catalyst; however, it requires photochemical excitation and proceeds via a radical pathway since it is not electrophilic enough to directly form the phosphine adduct [131, 132].

**FIGURE 21:**
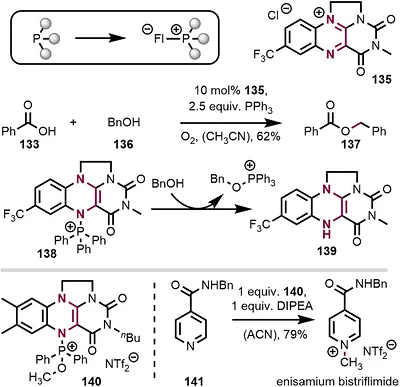
Covalent adducts with phosphines (top) and phosphinites (bottom), and their applications.

The reactivity of flavinium salts is not limited to phosphines as P‐nucleophiles. Our group reported the addition reaction with phosphinites, yielding adduct **140**. The latter is an active methylation agent, which was, inter alia, capable of converting pyridine **141** into enisamium bistriflimide [133]. Here, the hydroquinoid flavin mediator is easily recovered by acidic hydrolysis and re‐oxidation by air.

## 7 Brønsted Acid Photocatalysis

One particularly intriguing activity is the use of flavins as Brønsted acid photocatalysts (Figure 22). Arakawa and Imada reported on the activity of flavin semiquinones as mild acids in the formation of thioketals. The authors observed the efficient formation of thioacetal **144** by irradiation of a solution of 4‐methylbenzaldehyde (**142**) and propane‐1,3‐dithiol (**143**) in the presence of **RFTA** [134]. Based on their mechanistic analysis, the authors proposed the formation of protonated semiquinone (**RFTA‐H** ^•^), which was reported to exhibit a p*K*_a_ of 8.3 in aqueous solution [135, 136]. The Bloom group also used the Brønsted acidity strategy. However, they generated the excited protonated **RFTB** by photo‐electrochemistry [137]. This species was characterized by an even stronger acidity (p*K*_a_ = −6) [138], which allowed the authors, *inter alia*, to facilitate a Ritter reaction converting ester **145** to amide **146**.

**FIGURE 22:**
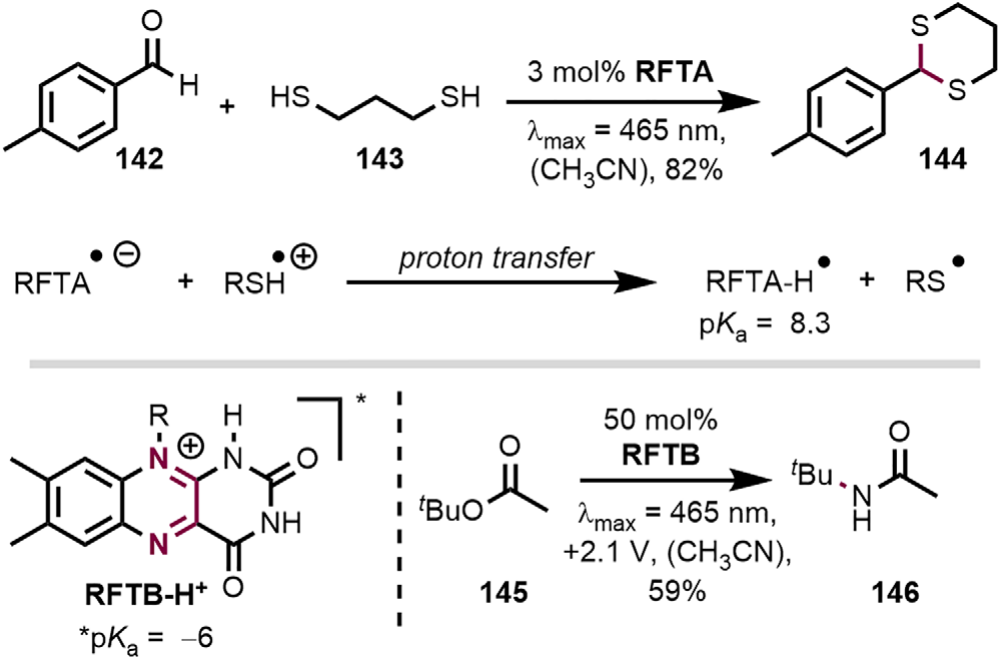
Flavins as Brønsted acid catalysts. Photoreduction of a flavin catalyst with concomitant proton transfer furnishes its neutral, acidic radical (top). An electrochemically generated riboflavin tetrabutyrate cation catalyzes a Ritter reaction upon excitation (bottom).

## 8 Challenges and Conclusion

Flavins and their congeners—such as alloxazines and deazaflavins—in different oxidation states have enjoyed a resurgence as homogeneous catalysts over the last decade, demonstrating reactivity not only in ground state catalysis via covalent adducts but also in photochemical oxidations/reductions and sensitization. Most reactions share a biomimetic nature of sorts with many transformations inspired by well‐studied enzymatic mechanisms. Applications greatly benefit from the catalysts’ stability in their respective redox and excited states as well as from balanced dissociation energies of their covalent adducts allowing for bond formation/dissociation events, mimicking pathways established in nature over millenia as cofactors in active sites.

In the field of flavin catalysis, several challenges remain. One of these involves the unambiguous characterization of transient flavin intermediates in catalytic cycles. Such species—including semiquinone radicals, covalent adducts, and hydroperoxides—are part of many synthetic and natural transformations. A detailed understanding of their stability and follow‐up reactions is needed to, for example, reduce the catalytic loading and increase turnover numbers. Additionally, fundamentally new avenues of reactivity will likely spawn from a deeper understanding of the reactivity of reactive (excited) flavin species, especially their chemically modified analogues. Building on the biomimetic nature of the field, chemically designed small‐molecule dimeric or oligomeric binding pockets with specific substrate affinities are envisioned to pave the way for exciting stereo‐ and site‐selective flavin catalysis in the organic laboratory.

## Author Contributions

**Jan Freudenberg**: conceptualization, writing – original draft, visualization, writing – review and editing. **Golo Storch**: writing – original draft, funding acquisition, conceptualization, writing – review and editing, visualization.

## Conflicts of Interest

The authors declare no conflicts of interest.

## Data Availability

Data sharing is not applicable to this article, as no new data were created or analyzed in this study.
